# A Potential Driver of Disseminated Intravascular Coagulation in Heat Stroke Mice: Neutrophil Extracellular Traps

**DOI:** 10.3390/ijerph191912448

**Published:** 2022-09-29

**Authors:** Yuling Zhang, Xiling Deng, Jing Zhang, Liang Zhang, Zubair Akram, Bo Zhang, Shiguo Sun

**Affiliations:** 1 Key Laboratory of Xinjiang Phytomedicine Resource and Utilization, Ministry of Education, Shihezi University, Shihezi 832000, China; 2 Key Laboratory for Green Processing of Chemical Engineering of Xinjiang Bingtuan, School of Chemistry and Chemical Engineering, Shihezi University, Shihezi 832000, China; 3 College of Chemistry and Pharmaceutical Engineering, Hebei University of Science and Technology, Shijiazhuang 050000, China

**Keywords:** heat stroke, neutrophil extracellular traps, disseminated intravascular coagulation

## Abstract

**Highlights:**

**What are the main findings?**

- NETs contribute to the activation of the coagulation cascade and have been successfully proved to be a potential driver of DIC in HS mice.

**What is the implication of the main finding?**

- This work provides a novel alternative treatment strategy for the treatment of DIC in HS patients.

**Abstract:**

Aims: Disseminated intravascular coagulation (DIC) is a common complication of heat stroke (HS) patients, and it is one of the important reasons leading to multiple organ failure and even death. The association between neutrophil extracellular traps (NETs) and DIC is unclear in HS mice. Methods and results: Here, HS was induced by the combination of hyperthermia (HT) and lipopolysaccharide (LPS). The DIC was evaluated by measuring prothrombin time (PT), D-dimer, thrombomodulin (TM), fibrinogen (FIB), and platelet (PLT). The expression of citrullinated-histone (CitH3) was analyzed by Western blotting. The formation of NETs was observed by immunofluorescence microscopy. The risk of HS-induced DIC was increased when HT was combined with LPS. The markers of NETs were significantly higher than those in the control group, and the NETs derived from HS promoted the development of DIC. DNase I improved coagulation dysfunction via the clearance of NETs caused by neutrophil aggregation. Conclusions: Degradation of NETs reduced the risk of developing DIC, and thus the survival rate of mice was improved. These results indicate that NETs may hold potential alternative therapeutic strategies for the treatment of DIC in HS patients.

## 1. Introduction

Disseminated intravascular coagulation (DIC) is a common and life-threatening complication of heat stroke (HS)—it is defined clinically as a core temperature (Tc) > 40.6 °C, which is characterized by activation of the coagulation system [1,2]. DIC can increase the mortality of HS patients, resulting in a huge medical burden. The symptoms caused by HS are similar to those caused by infection [3,4]. Heat stress increases the permeability of the gastrointestinal tract and makes the endotoxin of the gastrointestinal flora enter the bloodstream, resulting in endotoxemia. Therefore, HS is regarded as “sepsis-like” [5]. Similarly, pre-existing viral illness or lipopolysaccharides (LPS) can increase the risk of HS, which may be related to coagulation dysfunction and immune function impairment [6,7,8]. Although the presence of standard and supportive treatment, most HS patients may show DIC leading to death [1,9]. It is generally believed that DIC is caused by the release of tissue factor into the bloodstream after vascular wall injury, which leads to the generation of thrombin, and then conversion of fibrinogen (FIB) to fibrin [10]. Notably, recent experimental studies have shown that various components of neutrophil extracellular traps (NETs) are also considered blood coagulation activators, which can activate the formation of platelet count (PLT) in vitro and participate in the blood coagulation process of endogenous and exogenous pathways [11,12,13,14]. However, the role of NETs in the development of DIC induced by HS has not yet been demonstrated.

NETs, a mechanism of neutrophil-killing microorganisms, were proposed by Brinkmann et al. in 2004 [15]. NETs contain circulating cell-free DNA (cfDNA), CitH3, and granule proteins [16]. NETs have been described as a double-edged sword. The uncontrolled or excessive release of NETs may cause injury to the surrounding cells, leading to NETs-associated diseases [17]. In previous studies, NETs played a vital role in the procoagulant activity and thrombotic tendency, and DNAse I can reduce procoagulant activity by inhibiting the formation of NETs [18,19,20,21]. Mechanically, DNA in NETs can activate the extrinsic coagulation pathway, while NETs provide tissue factors to initiate the intrinsic pathway [13,22]. In addition, histones in NETs can act as ligands of Toll-like receptors on PLT, which can promote PLT activation, and activated PLT further enhance the formation of NETs [23,24]. Therefore, we believe that targeting NETs may similarly reduce the risk of DIC in HS mice.

Based on the above findings, the purpose of this study is to elucidate the potential role of NETs releases in HS-associated DIC. The present research results might identify the role of NETs in the onset of HS and help to explore the clinical potential target for HS.

## 2. Methods

### 2.1. Animals

All experiments were conducted with Kunming (KM) male mice age-matched 10–12-week old (20–22 g), which were obtained from animal house medical laboratory, Xinjiang Medicine University (Xinjiang, (SCXK (Xin) 2016–0003), China). In total, 96 mice were housed individually at an ambient temperature (Ta) of 25 ± 1 °C, relative humidity of 40 ± 5%, and a 12-hour light/dark cycle for 1 week prior to the start of the experiments (lights on at 7:00 A.M. and off at 7:00 P.M.). Pelleted mice feed and tap water were provided ad libitum. The experimental protocol was approved by the Ethics Committee at The First Affiliated Hospital of Medical College of Shihezi University (approval nos: A2018-100-01) in accordance with the guidelines of the US National Institutes of Health.

### 2.2. Establishment of HS Model

Forty mice were randomly divided into four groups: control (CTR) group, hyperthermia (HT) group, hyperthermia and lipopolysaccharide stress (HT + LPS) group, and lipopolysaccharide (LPS) group. In HT + LPS group and LPS group, 10 mg/kg LPS (1 mg/mL) was injected via tail vein, and in the CTR group and HT group, 0.9% normal saline was injected into the tail vein. In the HT group and HT + LPS group, ambient temperature was 41.0 ± 1 °C, and relative humidity was maintained 55 ± 5%. In the CTR group and LPS group, the ambient temperature was 25.0 ± 1 °C, and relative humidity was 40 ± 5%; LPS (from *Escherichia coli*) was injected slowly through the tail vein for 10 min and then transferred to an artificial climate box (Ningbo Jiangnan Instrument Factory, Ningbo, China) for heat exposure. Tc was monitored using an electronic thermometer and connected to the BL-420/820 biological signal acquisition and processing system. Tc, heart rate (HR), and respiratory rate (RR) were recorded every 15 min until Tc ≥ 42.4 °C. Immediately after the onset of HS, mice were removed from the box and allowed to attain room temperature (25.0 ± 1 °C) in a new cage with food and water ad libitum. Mice were fasted and water-free for 12 h before the experiment.

### 2.3. Diagnostic Criteria for Experimental HS and DIC

The diagnosis of HS refers to clinical diagnostic criteria because of the different judgment criteria in experimental animals (the Tc is more than 40 °C, and the patients with consciousness disorders are the diagnostic standards) [5,6,25,26]. In this study, Tc ≥ 42.4 °C was maintained, the time point of arrhythmia in mice as a marker of the onset of HS.

Guidelines for the diagnosis and management of DIC [27] and the diagnostic criteria of the animal DIC model established by Kessler [28] were adopted.

### 2.4. Biochemical and Cytokines Measurement of Blood

When HS occurred, blood from each group was collected into tubes containing anticoagulant by eyeball blood sampling and centrifuge at 3500 rpm for 15 min at 4 °C. No anticoagulant was added when collecting the serum; other conditions were the same. Except for the HT + LPS group, all other groups were euthanized at 90 min of heat stress. The aseptic operation was maintained throughout the experiment. The plasma levels of PT, thrombin time (TT), activated partial thromboplastin time (APTT), and FIB was determined by an Automatic coagulation analyzer (ACL-TOP700, Instrumentation Laboratoy company, Boston, MA, USA). The plasma levels of white blood count (WBC) and PLT were determined by an Automated hematology analyzer (XT-2000i, Sysmex, Kobe, Japan). EDTA was used for PT, TT, APTT, and FIB analysis, and heparin was used for WBC and PLT analysis. The serum levels of lactate dehydrogenase (LDH), Creatine kinase (CK), aspartate aminotransferase (AST), alanine aminotransferase (ALT), blood urea nitrogen (BUN), and creatinine (CRE) was determined by the assay kit (Nanjing Jiancheng Bio-Tech Co., Ltd., Nanjing, China) according to manufacturer’s instructions. The plasma levels of D-dimer, CitH3, and TM were measured using commercially available ELISA kits according to the manufacturer’s instructions. (D-dimer and TM from Westang Bio-Tech Co., Ltd., Shanghai, China; CitH3 from Cohesion, Kent, UK). The coagulation time (CT) was measured by using the capillary method (CT refers to the time required for coagulation of isolated venous blood, reflecting the function of endogenous coagulation pathways). Store the plasma and serum at −80 °C until further use.

### 2.5. Histological Examination

Tissue samples of heart, liver, and kidney were collected immediately following blood withdrawal and fixed in 4% paraformaldehyde solution. All the preserved tissues were embedded in paraffin, cut into sections (5 μm) with a microtome, followed by staining with hematoxylin and eosin (H&E). Subsequently, stained specimens were imaged under a light microscope with a digital camera system (Axio Imager M2 microscope, Carl Zeiss, Oberkochen, Germany).

### 2.6. Neutrophil Isolation and NETs Production

Neutrophils were obtained from fresh heparinized peripheral blood of healthy mice. Neutrophils were isolated by Percoll (GE Healthcare) gradient centrifugation, followed by hypotonic lysis of residual erythrocytes [29]. Isolated neutrophils were washed with PBS before use. Peripheral blood-derived neutrophils were isolated, as previously described. Purified neutrophils were suspended in Dulbecco’s Modified Eagle’s Medium (DMEM) at a concentration of 1.0 × 10^7^ cells/mL. NETs were prepared as previously described [24,30]. In brief, to generate NETs, isolated neutrophils were resuspended in DMEM medium supplemented with 3% fetal bovine serum (NET medium), and 2.0 × 10^6^ neutrophils per well were seeded on 24 well plates. The cells were activated with different groups of plasma for 4 h in 5% CO_2_ at 37 °C. These plasmas were from the CTR group, HT group, HT + LPS group, and LPS group, respectively.

### 2.7. Western Blot Analysis

The expression of CitH3 in neutrophils was detected by Western blotting. Protein concentration was determined by the BCA assay (Beyotime, Shanghai, China). CitH3 was labeled with primary Anti-Histone H3 antibody (citrulline R2 + R8 + R17, CPA9347, Cohesion, Kent, UK; dilution 1:1000) and secondary Goat anti-rabbit IgG (ab150077, Abcam, Cambridge, UK, dilution 1:10,000). Densitometry on western blots carried out using Image J software (National Institutes of Health). In brief, cells were homogenized in an ultrasonic cell disruptor (Ningbo Scientz Biotechnology Corporation, Ningbo, China) and then incubated on ice for 30 min, centrifuged at 12,000 rpm at 4 °C for 10 min. The protein concentrations were measured with Quawell Q5000 micro-volume UV-Vis spectrophotometer, measuring absorbance ratios of 260 nm/280 nm. (Aidlab Biotechnologies Co., Ltd., Beijing, China). The samples were incubated with Anti-Histone H3 (1:1000). After washing (TBST buffer, 150 mM NaCl, 10 mM Tris pH 7.5, 0.1% Tween 20), membranes were incubated with secondary Goat anti-rabbit IgG for 1–2 h at room temperature. The blots were stained using the SuperSignal WestPico Chemiluminescense substrate (Thermo Scientific, Rockford, IL, USA), and protein levels were quantified using a UVP EC3 imaging system (Upland, CA, USA).

### 2.8. NETs Quantification and Image Analysis

Antibacterial peptide-37 (LL-37), cfDNA, and CitH3 have been studied as surrogate markers for NETs releasing [15,31,32]. Plasma levels of cfDNA were measured using a fluorometric assay for double-stranded DNA Quant-iT PicoGreen^®^ dsDNA Assay kit (Solarbio Science-Tech Co., Ltd., Beijing, China) following the manufacturer’s instructions. The amount of DNA was reflected by the fluorescence intensity, which was measured at excitation and emission wavelengths of 480 nm and 530 nm, respectively. Plasma levels of LL-37 were determined by ELISA with Mouse LL-37 ELISA Kit (Shuangyin Bio-Tech Co., Ltd., Shanghai, China). Fluorescence imaging of NETs components was performed with immunofluorescence analysis. Extracellular DNA was labeled with Hoechst 33258 (E607329, Sangon Biotech, Shanghai, China, 5 μM), and CitH3 was labeled with the primary Anti-Histone H3 antibody (citrulline R2 + R8 + R17, CPA9347, Cohesion, Kent, UK; dilution 1:800) and secondary Alexa Fluor 488-conjugated Goat anti-rabbit IgG (ab150077, Abcam, Cambridge, UK; dilution 1:1000). Data are expressed as the percentage of area covered by positive fluorescence staining for each field of view.

### 2.9. Statistical Analysis

All results were expressed as mean ± standard deviation (SEM) and were analyzed using SPSS 20.0 (IBM SPSS, Armonk, NY, USA) and GraphPad Prism 7 (GraphPad Software Inc., La Jolla, CA, USA). Data were analyzed by one-way analysis of variance (ANOVA) with Fisher’s LSD test. Survival curves were plotted by the Kaplan–Meier method and compared by the log-rank test. *p* < 0.05 was considered to indicate a statistically significant difference.

## 3. Results

### 3.1. HT + LPS Stress Increases the Risk of HS-Induced DIC

**Figure 1:**
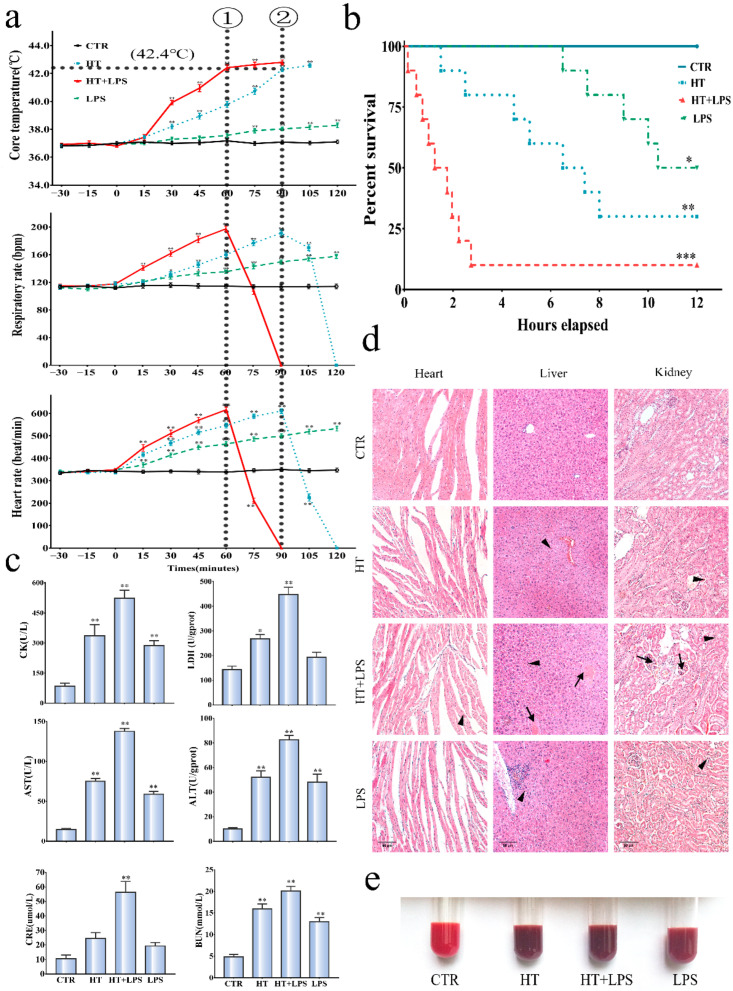
Effect of HT + LPS stress on HS model. (**a**) Effects of HT + LPS stress on vital signs. ①: Time of onset of HS in HT + LPS group; ②: Time of onset of HS in HT group. (**b**) Effects of HT + LPS stress on survival rate; the data were expressed as starting points from the onset of HS, which was monitored for 12 h (*p* < 0.05). (**c**) Effect of HT + LPS stress on organ damage in mice. (**d**) Effect of HT + LPS stress on heart, liver, and kidney histology. Black arrows point to hemorrhage. Black arrowheads point to thrombi. (**e**) Color contrast of peripheral blood in mice. Values are shown as the means ± SEM. * *p* < 0.05, compared with control group; ** *p* < 0.01, compared with control group, *** *p* < 0.001, compared with control group. H&E at magnification ×200.

HT and LPS caused significant changes in vital signs, survival rate, organ damage, and coagulation function in mice (vs. control group, respectively), as shown in Figure 1 and Table 1. When mice were transferred from normal temperature to an artificial climate box, the time point of arrhythmia and Tc reached above 42.4 °C was defined as the time point for the onset of HS; after 90 and 60 min of heat stress, HT group and HT + LPS group reached the time point of HS respectively (Figure 1a, *n* = 10 per group); In addition, the RR of HT group reached the highest at 90 min, and that of HT + LPS group reached the highest at 60 min. Similarly, the HR of the HT group reached the highest at 90 min, while that of the HT + LPS group reached the highest at 60 min, which were 610 ± 10.5 beat/min and 615.6 ± 11.2 beat/min, respectively. The survival curves derived using the Kaplan–Meier method are shown in Figure 1b (*n* = 10 per group), and 100% survival was observed in the CTR group during an experimental period of 12 h. When compared with the CTR group, the HT group had 30% survival with 12 h, the HT + LPS group was 10%, and the LPS group was 50%. Additionally, compared with the survival time of the HT group, the survival time of the HT + LPS group had significantly shorter (no more than 3 h). The levels of biomarkers of some organ damage in serum were detected (Figure 1c, *n* = 6 per group).

These markers represent heart injury (LDH, CK), liver injury (ALT, AST), and kidney injury (BUN, CRE), respectively. Significant differences were observed in serum levels of LDH, CK, ALT, AST, BUN, and CRE in the CTR, HT, HT + LPS, and LPS groups. The serum levels of ALT, AST, BUN, CRE, LDH, and CK in the HT + LPS group were significantly increased compared with the CTR group. The results showed that combined stress can aggravate organ damage more than single stress; The histopathological findings revealed the heart, liver, and kidney in the CTR group were unremarkable. In contrast, the damage noted in multiple organs in the model groups was extensive or relatively mild, respectively. Damage manifested as hemorrhage, thrombosis, increased inflammatory cells, and disruption of architecture. (Figure 1d, *n* = 6 per group). Moreover, the changing state of mice was described in Supplementary Materials (Figure S1, Supporting Information). The results further showed that combined stress can aggravate organ damage more than single stress; In addition, as shown in Figure 1e, compared with the CTR group, it was not difficult to find that the blood of the HT group and HT + LPS group was dark red, which indicated that coagulation occurred in the model group mice.

HS-induced DIC was evaluated by coagulation markers, namely PT, APTT, D-dimer, and FIB. Compared with the CTR, HT and HT + LPS stress induced coagulation activation, such as prolonged PT, APTT, elevated D-D and TM, and decreased PLT (Table 1, *n* = 6 per group). However, WBC was increased in the HT group and significantly decreased in the HT + LPS group. Moreover, previous studies also showed that WBC would be significantly reduced in the stage of severe HS [5]. PLT and FIB decreased rapidly, indicating that the hemostatic response to HS was not fully compensated. Histopathologic findings disclosed characteristic features of DIC, namely diffuse hemorrhage at focal sites in tissue from many organs, including the heart, liver, and kidneys, and widespread intravascular thrombi in small and medium-sized arteries and veins (Figure 1d). Thrombus formation was widespread in hepatic and renal veins in the HL + LPS group. The data indicated that HT + LPS stress can aggravate coagulation disorders, causing the formation of DIC.

### 3.2. HT + LPS Increases the Expression of NETs

**Figure 2:**
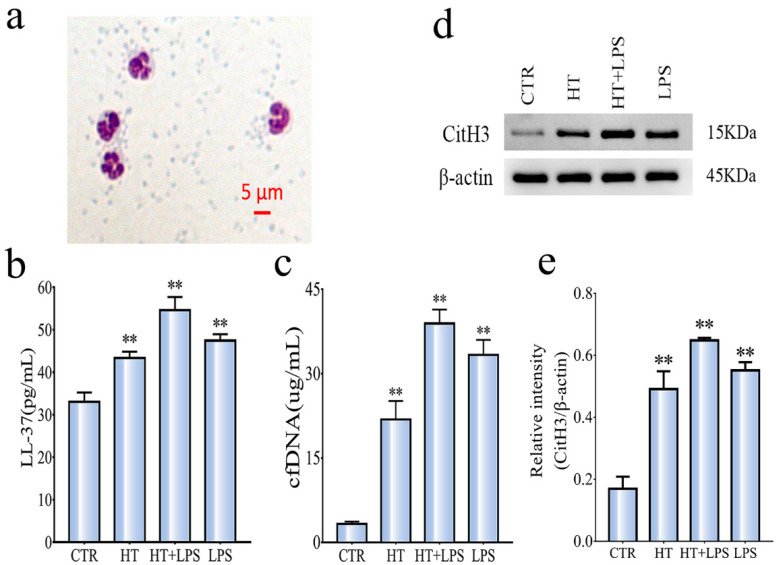
Effect of HT + LPS stress on HS model. (**a**) Morphologically by Diff-Quik staining and observation of cell morphology under optical microscope. (**b**,**c**) The level of NETs-related markers in plasma. (**d**) The expression of Cit-H3 protein. (**e**) Western blotting analysis of CitH3 in neutrophils among groups (*n* = 6 per group). Data are shown as mean ± SEM. ** *p* < 0.01, compared with control group.

The morphology of neutrophils after separation and purification is shown in Figure 2a, and the lobular nucleus was clearly distinguishable. LL-37 and cfDNA are two indirect plasma markers reflecting the level of NETs, which have indirectly reflected the formation of NETs in vivo [31,32]. Figure 2b,c (*n* = 6 per group) showed the plasma indirect marker of NETs, which was consistent with the results of Western blotting. NETs have been identified as extracellular structures composed of DNA and CitH3 [15,16]. The expression level of CitH3 protein in neutrophils of mice in each group was shown in Figure 2d. Compared with the CTR group, the expression level of CitH3 protein in the HT + LPS group was the highest, followed by the LPS group and then the HT group, indicating that HT + LPS have aggravated the expression of NETs in neutrophils of mice. Therefore, in subsequent experiments, we used the HT + LPS stress group as the HS model (HS).

### 3.3. Associations between NETs Markers and Coagulation Biomarkers in HS Mice

**Figure 3:**
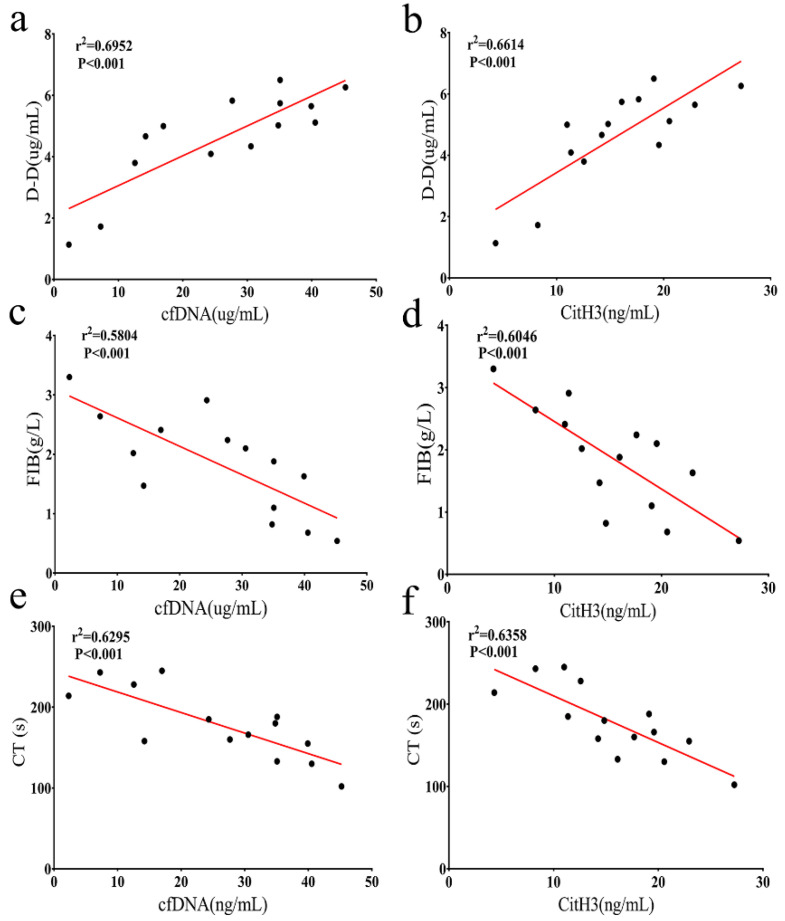
Correlation between NETs markers and hypercoagulable state in HS (*n* = 6 per group). (**a**,**b**), D-D was positively associated with circulating levels of cfDNA and CitH3. (**c**,**d**), FIB formation was inversely correlated with levels of cfDNA and CitH3. (**e**,**f**), CT was inversely correlated with levels of cfDNA and CitH3. CfDNA, cell-free deoxyribonucleic acid; D-D, D-dimer; CT, coagulation time; r values were assessed by Spearman rank correlation.

To study the effect of NETs on coagulation response during HS-induced DIC, CT, D-dimer (D-D), and FIB were measured. As shown in Figure 3 (*n* = 6 per group), D-D was positively correlated with circulating cfDNA and CitH3 levels in HS, and FIB was inversely correlated with levels of cfDNA and CitH3. Similarly, CT was inversely correlated with levels of cfDNA and CitH3. These results showed that NETs contribute to the activation of the coagulation cascade and that there is a greater risk of DIC in HS mice.

### 3.4. Procoagulant Activity of the NETs Generated from HS Neutrophils

**Figure 4:**
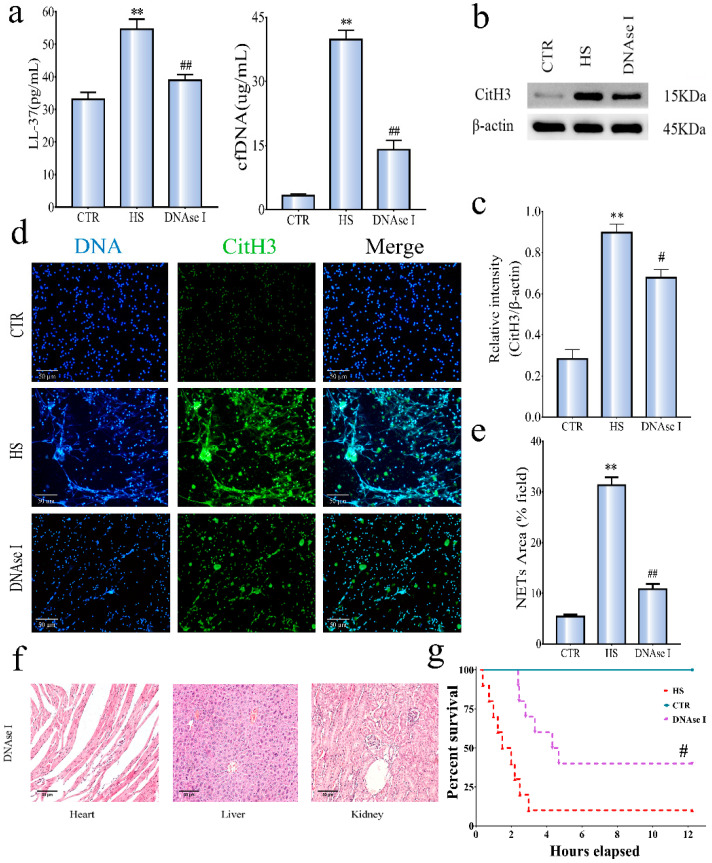
Effects of DNAse I on HS-induced NETs production. (**a**) The level of NETs-related markers in plasma. (**b**) The expression of Cit-H3 protein. (**c**) Densitometry of CitH3 vs. β-actin. (**d**) Representative image of immunofluorescence staining of NETs-related components (200×). (**e**) Quantitative analysis of NETs. (**f**) DNAse I-treated attenuates multi-organ injuries in mice during HS. (**g**) Effects of DNAse I on survival rate, the data were expressed as starting points from the onset of HS, which was monitored for 12 h. Extracellular DNA was in blue (Hoechst 33258), and CitH3 was shown in green (Alexa Fluor 488-conjugated goat anti-mouse H3). Scale bar: 50 μm. ** *p* < 0.01, vs. CTR. # *p* < 0.05, vs. HS; ## *p* < 0.01, vs. HS. H&E, hematoxylin and eosin; H&E at magnification ×200. Data are shown as mean ± SEM.

In order to further proved that intravascular coagulation activation was related to the existence of NETs, 2000 international units of DNAse I (i.v.) (a standard drug used for degradation of DNA in NETs [33]) were injected intravenously after the onset of HS (42.4 °C). Particularly, the administration of DNAse I did not produce any significant changes in normal mice (Figure S2). When HS mice died, tissue samples of each group were collected for H&E. The levels of LL-37 and cfDNA are shown in Figure 4a. Compared with the CTR group, the NETs markers released in the HS group increased significantly; on the contrary, the DNAse I treatment group significantly reduced the level of NETs markers. The expression of the CitH3 protein is shown in Figure 4b. In Figure 4c, Relative CitH3/β-actin activity among groups. By immunofluorescence staining, as shown in Figure 4d (*n* = 3 per group), HS caused neutrophils to release NETs, but these structures were not found in the CTR group. Compared with the HS group, DNAse I treatment reduced NETs release. In Figure 4e, the decrease in NETs can be seen under the treatment of DNase I. This also indicated that neutrophils would not release NETs without stimulation.

Tissue damage and thrombosis were markedly reduced in the liver, and kidney tissue from the DNAse I-treated HS group (Figure 4f) compared to the HS group. Table 2 (*n* = 6 per group) showed DNAse I had improved DIC induced by HS. Compared with the HS group, the levels of PT, TT, D-D, and TM in DNAse, I treatment group were significantly decreased, and the levels of PLT and FIB were significantly increased. As shown in Figure 4g, 100% survival was observed in the CTR group during an experimental period of 12 h. When compared with the CTR group, the HS group had 10% survival with 12 h, and the DNAse I group was 40%. The increase in the survival rate of DNAse I-treated mice was significant (*p* < 0.05). These data showed that NETs contribute to the activation of the coagulation cascade, and the treatment with NETs has improved HS-induced DIC.

## 4. Discussion

The current study showed that the HS model induced by HT combined with LPS increased the risk of DIC, and neutrophils from this model were more likely to form NETs. In addition, we have demonstrated that DNAse I treatment following HS alleviates DIC via degrading NETs and improves the survival rate of mice. To clarify the effect of NETs on DIC caused by HS, by establishing the mouse model of HS caused by HT combined with LPS, we tested the DIC-related indexes and NETs-related indexes. Once again, we proved that NETs can promote coagulation dysfunction. Our results suggested that NETs treatment can be a novel alternative treatment strategy for the treatment of DIC in HS patients.

HS is the most hazardous condition in a spectrum of illnesses progressing from heat exhaustion to heat stroke, characterized by a rapidly increasing Tc (Tc ≥ 40 °C). Hyperactivity of the coagulation system is very common in HS, with DIC as the first symptom [2]. Once patients develop DIC, the risk of death is significantly increased due to its unclear pathophysiology. Previous animal studies have shown that pre-existing inflammatory state (caused by exogenous administration of LPS) greatly aggravates the overproduction of proinflammatory cytokines, hypercoagulable state, and multiple organ injury, which induces the occurrence of HS [8]. In this study, we found that combined stress could not only aggravate the hypercoagulability of HS mice but also increase the risk of DIC. In addition, as shown in Table 1, the significant leukopenia caused by combined stress can be attributed to neutropenia, which was consistent with the results reported on severe HS in previous studies [5,34]. Therefore, we further explored the release of NETs in neutrophils under various stress states and found that combined stress significantly increased the release of NETs. In view of this phenomenon, the HT combined with LPS stress was used as the HS model in the present study to explore the correlation between the release of NETs and DIC in HS.

NETs were initially considered to be the complex function of the innate immune system against invading microorganisms, but researchers gradually realized that NETs have farther-reaching capabilities. On the one hand, NETs capture pathogens (such as bacteria and fungi) to fix and eliminate them, which is an important mechanism for eliminating pathogens [15]. On the other hand, it may have a serious impact when the generation or clearance of NETs is inadequately controlled [14,17,35,36,37]. Previous studies have suggested that the pathophysiological mechanism of HS-induced DIC is excessive coagulation activation and fibrin formation, such as the massive consumption of platelets and the increase in D-dimer and thrombin levels [9,38,39]. In recent years, it has been displayed that DNA and citrullinated histones in NETs are related to coagulation dysfunction [12]. However, the role of NETs has not been reported in HS. Here, we found that the levels of NETs markers in the HT group and HT + LPS group were significantly higher than those in the CTR group, and the formation of thrombosis in the HT + LPS group was more obvious, as shown in Figure 1 and Figure 2. In addition, bidirectional interactions between NETs and platelets may also be critical for HS-related thrombosis, which has been demonstrated in a variety of disease models [12,22]. Similarly, it was also revealed in our study that NETs in HS significantly enhanced the procoagulant activity, resulting in an increase in FIB level and the shortening of CT (Figure 3). Therefore, these results indicate that NETs are involved in the disease progression of HS.

In order to further clarify the role of NETs in HS-induced DIC, we used DNAse I to disassemble the NETs and found that DIC-related indicators also decreased (evidenced by decreased levels of PT, D-dimer, TM, and increased levels of PLT counts), as shown in Figure 4 and Table 2. Similarly, by testing the survival rate of mice, as shown in Figure 4g, we found that the survival rate of mice after DNA treatment was significantly improved. It is worth noting that prolonging the survival time of HS patients will help them to obtain effective treatment. Since DNAse I is a standard drug used for the degradation of DNA in NETs, it is suggested that DNase I improves DIC by degrading NETs. Therefore, our research shows that the abnormal expression of NETs will aggravate the risk of DIC in HS.

This study has several limitations. First of all, although we try to simulate clinical HS patients as much as possible, the difference from the actual clinical situation is that HS patients cannot be treated immediately after heat stress. Secondly, the potential roles of proinflammatory cytokines (especially TNF- α and IL-1 β) and chemokines were not studied, which is a limitation of this study. In addition, DNase I can only degrade existing NETs but cannot inhibit the formation of NETs, which may be further verified in future work. Finally, since there are two ways to form NETs-classical NETosis and vital NETosis [40], we will still need to consider how HS produces NETs in future research, which may play an important role in the treatment of HS.

## 5. Conclusions

In conclusion, this study showed that HS caused neutrophils to release NETs in mice. The overexpression of NETs in HS mice will increase the risk of DIC. In addition, the degradation of NETs can reduce the development of DIC in HS mice and improve the survival rate of HS mice. Therefore, it is demonstrated that NETs are a potential driver of DIC induced by HS, providing a mechanistic basis for NETs’ therapeutic potential in HS.

## Figures and Tables

**Table 1:** Effect of HT + LPS stress on coagulation biomarkers.

Variable	CTR	HT	HT + LPS	LPS
WBC (×10^9^/L)	3.3 ± 0.4	6.1 ± 1.0 **	1.4 ± 0.3 *	1.3 ± 0.5 *
PLT (×10^9^/L)	1019 ± 56	744 ± 32 *	453 ± 48 **	710 ± 75 *
PT (sec)	9.4 ± 0.3	12.4 ± 0.3 **	16.7 ± 0.6 **	10.3 ± 0.2
APTT (sec)	22.4 ± 0.5	27.5 ± 0.5	32.8 ± 1.2 **	23.9 ± 2.3
TT (sec)	13.5 ± 0.2	17.2 ± 1.0 *	23.6 ± 1.3 **	15.2 ± 0.6
FIB (g/L)	2.1 ± 0.1	1.9 ± 0.1	1.4 ± 0.2 *	2.3 ± 0.1
D-D (μg/L)	25 ± 3	104 ± 10	6036 ± 130 **	5546 ± 250 **

Values are shown as the means ± SEM (*n* = 6). * *p* < 0.05, compared with control group; ** *p* < 0.01, compared with control group. WBC, white blood count; PLT, platelet count; PT, prothrombin time; APTT, activated partial thromboplastin time; TT, thrombin time; FIB, fibrinogen; D-D, D-dimer.

**Table 2:** Effect of DNAse I on coagulation biomarkers.

Variable	CTR	HS	DNAse I
WBC (×10^9^/L)	3.3 ± 0.4	1.4 ± 0.3 *	1.9 ± 0.5
PLT (×10^9^/L)	1019 ± 56	453 ± 48 **	727 ± 57 #
PT (sec)	9.4 ± 0.3	16.7 ± 0.6 **	12.0 ± 0.6 #
TT (sec)	13.5 ± 0.2	23.6 ± 1.3 **	18.4 ± 1.1 ##
FIB (g/L)	2.1 ± 0.1	1.4 ± 0.2 *	2.0 ± 0.1 #
D-D (μg/L)	25 ± 3	6036 ± 130 **	5021 ± 213 ##
TM (μg/L)	174 ± 30	8532 ± 648 **	2902 ± 458 ##

Values are shown as the means ± SEM (*n* = 6 per group). WBC, white blood; PLT, platelet; PT, prothrombin time; TT, thrombin time; FIB, fibrinogen; D-D, D-dimer. TM, thrombomodulin. * *p* < 0.05, vs. CTR; ** *p* < 0.01, vs. CTR. # *p* < 0.05, vs. HS; ## *p* < 0.01, vs. HS.

## Data Availability

All data analyzed are included in this article and its online Supplementary Material files. Further enquiries can be directed to the corresponding author.

## Supplementary Materials

The following supporting information can be downloaded at: https://www.mdpi.com/article/10.3390/ijerph191912448/s1, Description S1: Physiological conditions of mice in each group; Figure S1: Changes in the state of mice in each group. (**a**,**c**) Changes of body features in mice; (**b**) The color changes of main organs in each group; (**d**) The weight changes of mice in each group; Figure S2: Effects of DNase I on normal mice. (**a**–**f**) Organ damage markers of mice in each group; (**g**) Changes of WBC; (**h**) Changes of PLT.

## Abbreviations

HT: Hyperthermia; HS, Heat Stroke; LPS, Lipopolysaccharides; CTR, Control; HT + LPS, Hyperthermia And Lipopolysaccharide Stress; HE (H&E), Hematoxylin-Eosin Staining; NETs, Neutrophil Extracellular Traps; CitH3, Citrullinated-Histone; cfDNA, Circulating Cell-Free DNA; HR, Heart Rate; RR, Respiratory Rate; AST, Aspartate Aminotransferase; ALT, Alanine Aminotransferase; BUN, Blood Urea Nitrogen; LDH, Lactic Dehydrogenase; CRE, Creatinine; DIC, Disseminated Intravascular Coagulation; CK, Creatine Kinase; DMEM, Dulbecco’s Modified Eagle’s Medium; PT, Prothrombin Time; APTT, Activated Partial Thromboplastin Time; TT, Thrombin Time; FIB, Fibrinogen; TM, Thrombomodulin; WBC, White Blood; PLT, Platelet; D-D, D-Dimer; CT, Coagulation Time; LL-37, Antibacterial Peptide-37; NE, Neutrophil Elastase; MPO, Myeloperoxidase.
