# Supplementary material for: A Potential Driver of Disseminated Intravascular Coagulation in Heat Stroke Mice: Neutrophil Extracellular Traps
Source: Int J Environ Res Public Health. 2022 Sep 29;19(19):12448. doi: 10.3390/ijerph191912448 (PMC9566744; doi:10.3390/ijerph191912448)
Supplement: Supplementary file 1 [file ijerph-19-12448-s001.zip › ijerph-1900076-supplementary.pdf]

A Potential Driver of Disseminated Intravascular Coagulation in Heat Stroke Mice: Neutrophil Extracellular Traps

Yuling Zhang [1,2], Xiling Deng [1], Jing Zhang [1,2], Liang Zhang [1], Zubair Akram [2], Bo Zhang [1,*] and Shiguo Sun [1,3,*]

[1] Key Laboratory of Xinjiang Phytomedicine Resource and Utilization, Ministry of Education, Shihezi University, Shihezi 832000, China
[2] Key Laboratory for Green Processing of Chemical Engineering of Xinjiang Bingtuan, School of Chemistry and Chemical Engineering, Shihezi University, Shihezi 832000, China
[3] College of Chemistry and Pharmaceutical Engineering, Hebei University of Science and Technology, Shijiazhuang 050000, China
* Correspondence: bozhang_lzu@126.com (B.Z.); sunsg@nwsuaf.edu.cn (S.S.)

Description S1: The mice in the CTR group had normal mental state, stable respiration, free movement, good diet, lustrous hair, no abnormal change in posture, and no diarrhea. In the HT group, the mice exhibited obvious symptoms such as wet body hair, red ears, mouth and feet, salivation in mouth and nose, and mouth-opening breathing. In the early stage of heat exposure, the mice were agitated and scurrying about, and in the later stage, the mice were depressed, tired and immobile, and had unstable gait. In addition, the onset of heat stroke included convulsion, dry feces mass, and decreased defecation. Except for the characteristics of fecal adhesion, turbid urine and diarrhea in the HT+LPS group, the other characteristics were generally consistent with the state of mice in the HT group. In the LPS group, the mice showed malaise, decreased activity, slow movement, upright back hair, chills, and viscous secretion in the eyelid. Dilute yellow feces and diarrhea appeared at the anus of the mice. As shown in supplementary Figure S1a and S1c. Figure S1b shows the

color changes of main organs in each group. The weight changes of mice in each group were shown in Figure S1d, among which the weight changes of mice in HT and HT+LPS groups were the most significant.

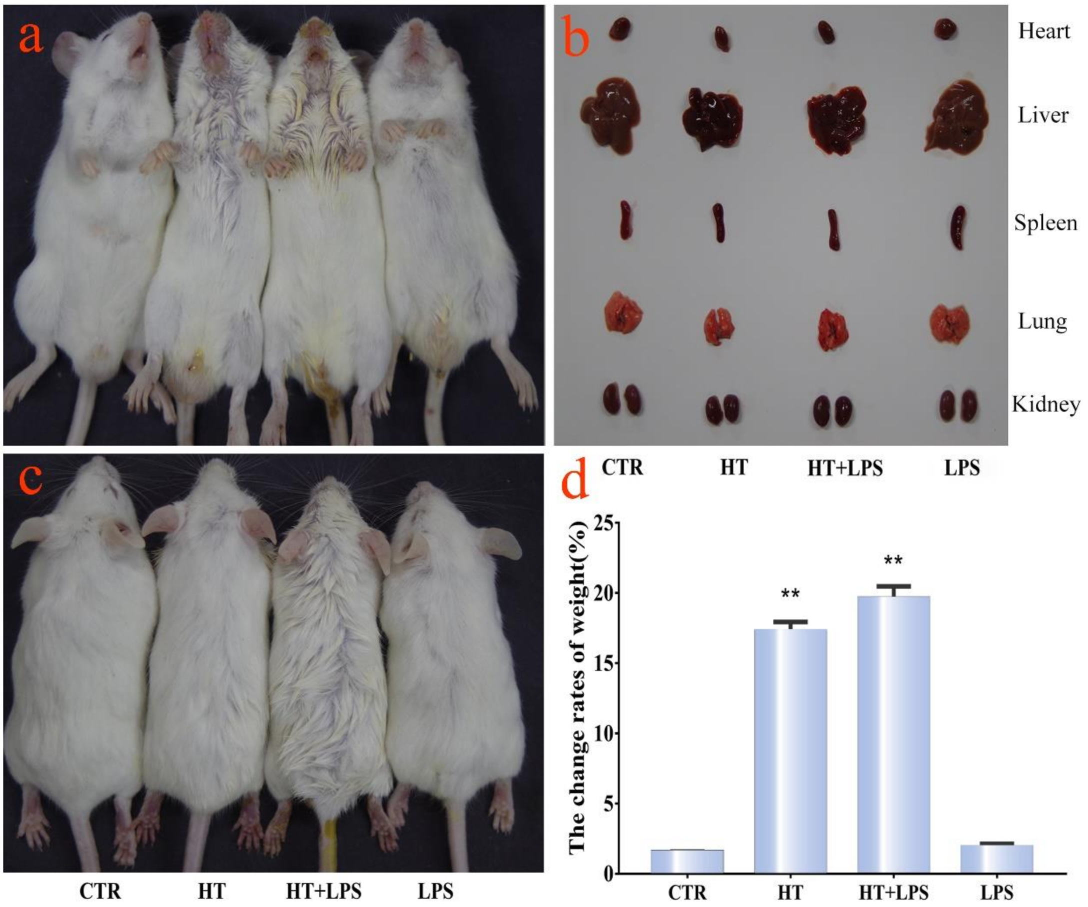

Figure S1. Changes in the state of mice in each group. (a,c) Changes of body features in mice; (b) The color changes of main organs in each group; (d) The weight changes of mice in each group. Data are presented as Mean ± SEM. $^{**}P < 0.01$, compared with CTR group.

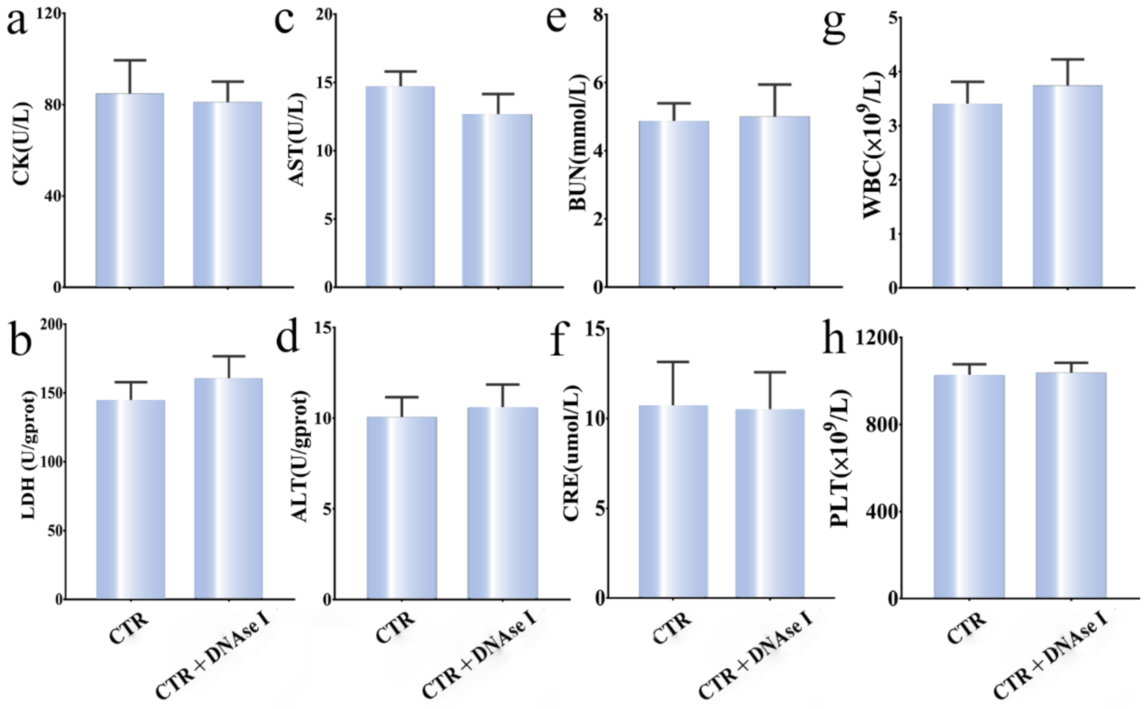

Figure S2. Effects of DNase I on normal mice. (a-f) Organ damage markers of mice in each group; (g), Changes of WBC; (h), Changes of PLT. Data are presented as Mean ± SEM. No significant intergroup differences were detected. N=6 mice per group.
